# Tools for Detection of Schistosomiasis in Resource Limited Settings

**DOI:** 10.3390/medsci6020039

**Published:** 2018-05-23

**Authors:** Olumide Ajibola, Bashar Haruna Gulumbe, Anthonius Anayochukwu Eze, Emmanuel Obishakin

**Affiliations:** 1 Department of Microbiology, Federal University Birnin Kebbi, P.M.B. 1157 Kalgo Road, Birnin Kebbi 860222, Kebbi State, Nigeria; olumide.ajibola@fubk.edu.ng (O.A.); hgbashar@gmail.com (B.H.G.); 2 Department of Medical Biochemistry, University of Nigeria, Enugu Campus, Enugu 400241, Enugu State, Nigeria; 3 Biotechnology Division, National Veterinary Research Institute, P.M.B. 001, Vom, Jos, 930281, Plateau State, Nigeria; emmanuel.obishakin@nvri.gov.ng

**Keywords:** diagnosis, schistosomiasis, neglected, Africa

## Abstract

Schistosomiasis is a debilitating disease affecting over 200 million people, with the highest burden of morbidity and mortality in African countries. Despite its huge impact on the health and socio-economic burden of the society, it remains a neglected tropical disease, with limited attention from governments and stakeholders in healthcare. One of the critical areas that is hugely under-developed is the development of accurate diagnostics for both intestinal and urogenital schistosomiasis. Diagnosis of schistosomiasis is important for the detection and treatment of disease in endemic and non-endemic settings. A conclusive detection method is also an indispensable part of treatment, both in the clinic and during mass drug administration (MDA), for the monitoring efficacy of treatment. Here, we review the available diagnostic methods and discuss the challenges encountered in diagnosis in resource limited settings. We also present the available diagnostics and cost implications for deployment in resource limited settings. Lastly, we emphasize the need for more funding directed towards the development of affordable diagnostic tools that is affordable for endemic countries as we work towards the elimination of the disease.

## 1. Introduction

Schistosomiasis is a common neglected tropical disease that is caused by parasitic trematodes of the genus *Schistosoma*. About 207 million people are infected world-wide; 93% of this population live in Sub-Saharan Africa (SSA) with the largest numbers in Nigeria (29 million) and Tanzania (19 million), respectively [1]. Democratic Republic of Congo and Ghana contribute 15 million cases each to the burden of schistosomiasis globally [2,3,4,5]. Schistosomiasis causes an immense negative impact on the health and socio-economic life of households in affected countries [1]. The most affected group of people in terms of morbidity and mortality are school age children (SAC) and young adults. Schistosomiasis is caused by six species *Schistosoma mansoni*, Schistosoma haematobium, Schistosoma japonicum, Schistosoma mekongi, *Schistosoma guineensis*, and *Schistosoma intercalatum* [6]. In SSA, the two major forms of schistosomiasis are caused by *S. mansoni* (intestinal schistosomiasis) and *S. haematobium* (urogenital schistosomiasis). Clinical manifestations of chronic disease results from host responses to schistosome eggs. *S. mansoni* and *S. japonicum* eggs most commonly lodge in the blood vessels of the liver or intestine, causing diarrhea, constipation, and hematuria. Chronic inflammation can lead to bowel wall ulceration, hyperplasia, and polyposis, and in cases of heavy infections, liver fibrosis and portal hypertension may develop. Transmission of schistosomiasis is by contact with fresh water snails that are infected with the parasite [7]. In endemic countries, control of schistosomiasis is primarily through the use of preventive chemotherapy with 40 mg/kg praziquantel, as advocated by the World Health Assembly in 2001 through resolution 54.19 [8]. Mass drug administration (MDA) campaigns are conducted through national control programmes (NCP), which ensure the distribution of drugs to groups that are at risk of infection. Usually, school age children that are enrolled in schools and identified as infected through parasitological diagnosis are the focus of MDA and are treated following World Health Organization (WHO) guidelines. A major challenge with MDA is that only SAC enrolled in school are covered by MDA, therefore omitting SAC that are not enrolled in school. In addition, due to the heterogeneous geographical distribution of disease, there could be areas where children are under-or-over-treated with praziquantel. Unavoidable contacts with sources of infection by people in under resourced settings, or people that practice occupations that place them at high risk of infection also poses a challenge in controlling schistosomiasis through MDA alone [9]. These challenges in control, in addition to the likelihood of development of resistance to praziquantel, emphasize the need to apply MDA in tandem with other control activities, such as snail control, in order to achieve tangible results. 

With increased access to MDA through the establishment of NCP in almost all endemic countries, there is a need to assess the current parasitological tools that have limitations, especially when assessing communities working towards elimination or with light infections [10,11,12]. The tools currently that are applied in parasitological diagnosis for low intensity infections are insensitive and more sensitive tools available might be cost prohibitory and difficult to use in resource limited settings. As control efforts become more effective, diagnosis with the gold standard, which is microscopy, becomes more challenging. For example, in communities that were recently treated with praziquantel, it is difficult to estimate the actual infection prevalence when compared to those not treated. This makes it important to review current diagnostics in resource limited settings and the challenges encountered. Here we review the diagnostic tools available in resource limited settings, especially SSA and the challenges that are encountered in their application. We also provide a comparative cost analysis of available diagnostic tools for schistosomiasis detection.

## 2. Clinical Presentations of Schistosomiasis

People that have visited, or live in Africa or the Middle east, are likely to be infected by *S. haematobium*, *S*. *mansoni,* or *S*. *intercalatum*/*S*. *guineensis* (Central Africa only), while subjects from Asia or South America are likely to be infected by *S*. *japonicum*, *S. mansoni*, or *S. mekongi* [13,14,15]. Acute infections present with temporary urticarial rash and sometimes with katayama fever [16]. Intestinal schistosomiasis may cause abdominal pain and bloody loose stool, which may occur with the enlargement of the liver (in some cases, also the spleen), accumulation of fluid in the abdominal cavity, and portal hypertension [15]. Hematuria is usually manifested in urogenital schistosomiasis, together with bladder and ureter fibrosis, and in advanced pathology, kidney failure [15]. *Schistosoma* infected females may also present with genital lesions, vaginal bleeding and nodules in the vulva [15]. Clinical presentations of *Schistosoma* in females may be confirmed by abdominal ultrasound (to establish pathology of liver and spleen in intestinal schistosomiasis) and pelvic ultrasound (to establish pathology of the bladder, ureter, and kidney in urogenital schistosomiasis) [13]. Biochemical markers of schistosomiasis include hypo-albuminemia, increased levels of urea, creatinine, and hypergammaglobulinemia [17]. Increased serum levels of types III and IV protocollagen peptide, P1 fragment of laminin, hyaluronic acid, and fibrosin may suggest severe hepatic fibrosis [17].

## 3. Schistosomiasis Detection Methods

Currently available diagnostic methods for schistosomiasis include those that are relying on stool and urine microscopy for parasite detection (Kato Katz and urine microscopy), serum antibodies, antigen detection, and the detection of DNA. Accurate, cost effective, and easy-to-use diagnostic tests are required for successful NCP in endemic areas [18].

## 4. Urine and Stool Microscopy

Kato Katz (KK) is the most universally applied test for intestinal schistosomiasis (such as *S. mansoni*, *S. japonicum*, and *S. mekongi*), and it involves microscopic examination of a thick fecal smear for eggs that are shed by the infecting helminth. KK provides a high diagnostic specificity in endemic areas where parasite loads are high, but the detection limits falls to low levels when being applied in non-endemic areas or in the diagnosis of infections at an early stage. KK is cheap, easy to use, and diagnostic sensitivity can be improved by the examination of two or more KK thick smears from the same stool sample, thus eliminating the need for re-sampling [19]. The Helmintex method for stool analysis of *S. mansoni* has also been tested in Brazil with more improved sensitivity and specificity than KK and point-of-care (POC)-circulating cathodic antigen (CCA), although it has yet to be tested in the field in Africa [20]. Another approach that is used in stool examination is the fecal concentration technique (FECT). FECT suspends the fecal sample in formalin and ethyl acetate, centrifuge, the suspension sediments the parasite material in a pellet, which can subsequently be mixed with saline (or stained with iodine) for microscopy [21]. Urogenital schistosomiasis is usually assessed by the microscopic examination of parasite eggs in urine samples. About 10 mL of urine is filtered, and the residue that is examined for parasite eggs following centrifugation. The number of eggs per 10 mL of urine is used to express infection intensity [22]. 

One of the pitfalls of microscopic examination of eggs in urine or stool is that this approach is not sensitive enough for monitoring the efficiency of praziquantel treatment during MDA campaigns. Schistosome eggs might still be present in urine or stool weeks after the adult worms have died, conversely young worms (schistosomula) and worms that temporarily stopped shedding eggs will not be detected [23]. Microscopy is also limited in sensitivity, because it takes about two months from the time of infection for the eggs to appear in stool or urine [13]. Hence, this method of diagnosis may prevent the benefits of early detection and the treatment with praziquantel [24]. It is a general recommendation that testing for schistosomiasis be repeated by follow up with two consecutive visits for increased accuracy, since none of the current diagnostic tests is absolutely accurate [25]. This recommendation is really cumbersome with respect to sample collection, manpower, and materials that are required for processing specimens. Also, stool samples require immediate analysis as soon as possible because they cannot be stored, and this adds to the difficulties of diagnosis in resource-poor settings, especially where repeat testing is required. Urine samples, on the other hand, are easier to handle since they can be filtered in the field and stored for subsequent laboratory analysis. Urine microscopy is also regarded as the gold standard for *S. haematobium* eggs detection in endemic areas. However, in areas of low transmission intensity, which were recently treated with praziquantel, young children with light infections or adults with chronic infections who usually excrete few eggs, urine microscopy is not sensitive. Microscopy sensitivity can be improved by repeated screening of the children, but this brings up the total cost of diagnosis for each person, manpower needs and resources, which might be unaffordable. This inability to carry out repeated testing will result in an underestimation of the true burden of infection, necessitating a more sensitive diagnostic and cost effective tool in such settings [11,26]. In schistosomiasis screening campaigns, hematuria has been found to be significantly associated with *S. haematobium* infection [27]. Macro- or micro-hematuria can be assessed using dipstick [28]. However, a large percentage of micro-hematuria in low prevalence areas may be from other causes that are unrelated to *S. haematobium* infection. 

## 5. Antibody Based Detection Methods

Antibody based methods include indirect immunofluorescence (IFAT), enzyme-linked immunosorbent assay (ELISA), indirect hemagglutination (IHA), and are designed to detect antibodies in the serum of infected people. The sensitivity of IFAT and IHA in detecting schistosome infection compares favorably with standardized western blot analysis, with specificity of IHA depending on the application of the right cut off values [29]. ELISA, using the recombinant protein RP26, can be used to bind serum IgG, specifically detecting acute *S. mansoni* infection with a low cross reactivity profile [30]. The serological assay, colloidal dye immunofiltration (CDIFA) assay gives cheap, fast, and simple detection of *S. japonicum* in serum with a low cross-reaction profile [31]. CDIFA therefore may have the potential to fill the demand for a POC diagnostic for use in the field without any instrumentation being required. Generally, serological assays have improved level of sensitivity when the right tools are employed, as compared to microscopy for schistosome diagnosis [32]. However, errors may arise from false positive results from non-infected subjects carrying antibodies from previous exposure to schistosomiasis [17], and cross reactions with antibodies from other helminth infections [16]. Antibody detection methods are limited by their inability to distinguish between *Schistosoma* species, are more sensitive at later stages of infection, and are unable to differentiate between recent or past infections. Applicability of antibody based detection methods in SSA has been limited due to these factors, especially in areas where the disease is highly endemic and people living in the area are likely to be re-infected following treatment.

## 6. Antigen Based Detection Methods

Detection of adult worms and egg antigens in serum or urine holds a lot of diagnostic potential; it may ultimately replace the traditional methods, and the antigens that are detected in urine or serum are either anodic (circulating anodic antigen) or cathodic (circulating cathodic antigen) [17].

## 7. Circulating Anodic Antigen Assay

Circulating anodic antigen (CAA) assay uses luminescent quantitative up-converting phosphor (UCP) reporter particles and a rapid user-friendly lateral flow (LF) test format. Hence, UCP-LF CAA is an ultra-sensitive quantitation test that detects all *Schistosoma* spp. in blood, urine, or any other body fluid [23,33]. In Zanzibar, UCP-LF CAA assay showed significantly higher sensitivity than single urine filtration for *S. haematobium* detection, indicating the potential application of this assay [34]. In mapping studies of schistosomiasis, UCP-LF CAA assay has proved useful in assessing prevalence of disease, monitoring drug efficacy during MDA campaigns and re-infection since serum CAA levels are expected to decline shortly after praziquantel (PZQ) administration [23]. Increasing the volume of urine sample that is needed for CAA test was found to increase the sensitivity and the accuracy of CAA assay for *S. mansoni* in urine [35]. In addition, the ability of CAA test to detect a single pair of worms (early stage infection), correlation of the test results (CAA levels) to egg production and worm burden makes this assay a true measure of schistosomiasis infection [23]. Applicability of this test is still limited in field settings, as it involved a centrifugation step, including other resources that are needed for carrying out analysis, which will bring up the cost and the required personnel.

## 8. Circulating Cathodic Antigen Assay

This is an antigen test that is used as a point-of-care-CCA (POC-CCA) diagnostic specifically for the detection of active *S. mansoni* infection in humans [36]. CCA test has been suggested to be more sensitive than KK test, even when compared with three KK thick smears; in primary-school children that were treated with PZQ, one POC-CCA was found to be more sensitive than six KK in monitoring and evaluating the efficacy of PZQ in parasite clearance. POC-CCA has the advantage of using urine samples instead of stool for analysis, thereby increasing the acceptability by patients and the applicability for epidemiological surveys in large populations [37]. When being used in combination with serology for *S. mansoni*, a 100% detection sensitivity was achieved when compared to 95.7% and 91.3% sensitivity by serology and POC-CCA, respectively [38]. POC-CCA has been standardized for testing *S. mansoni* in urine with no special equipment being required could be applied for the detection of other intestinal schistosomes, such as *S. mekongi* and *S. japonicum* [33,39,40]. CCA test sensitivity and versatility makes it useful for detecting schistosomiasis in non-endemic locations, and for use as a reference test for treatment evaluation. Results from POC-CCA might give errors from trace values (results that fall between positive and negative outcomes), but it has been suggested that treating trace values as positive values significantly increased the sensitivity of the assay with better reproducibility of disease prevalence [41]. However, studies on the use of POC-CCA for *S. haematobium* present conflicting results. A study by Sanneh et al. found POC-CCA less sensitive and is less specific than the urine filtration and dipstick methods in the diagnosis of *S. haematobium* infection in endemic areas [42], while Said et al. observed a significantly higher sensitivity in the diagnosis of both *S. haematobium* and *S. mansoni* by this method as compared to egg counts in urine and stool, respectively [43]. In addition, limited sensitivity and false positives have been reported when POC-CCA was applied in Brazil and in some parts of Africa. In a study that was carried out in Brazil, POC-CCA was shown to have limited sensitivity as a stand-alone diagnostic in areas with low infection intensities, suggesting its limitation in countries moving towards the elimination of the disease. Similar findings have been observed by Bezerra et al. [44] and Adriko et al. [45], where limited sensitivity was reported in low endemic settings. POC-CCA also has the limitation of being a qualitative test; hence, its interpretation is based on individual analysis that could introduce some form of bias. Together with hematuria test strips, CCA cassette testing could have limited application in the screening and mapping of *S. haematobium* [46]. Despite the reliability of the POC-CCA, the inability to detect *S. haematobium* the most prevalent parasite in SSA is a major limitation. The cost of approximately $1.75 per test is also unaffordable for most laboratories in resource limited settings, despite the ease of use and sensitivity for the detection of *S. mansoni*.

## 9. DNA Detection

DNA-based assays have recently gained popularity for the detection of infectious disease agents.

In the detection of schistosomes, PCR techniques have exhibited a promising degree of sensitivity and specificity. A glimpse of what the application of the new DNA technologies might ultimately achieve was first proposed and demonstrated with southern blotting and hybridization methods [47,48,49]. Recently, the use of molecular methods in the detection and characterisation of schistosomes have been perfected [50,51]. PCR has been used in detecting schistosomes in human serum, urine and faeces, water, snails, and many other sample types [47,52,53,54,55]. Amplification of cell-free DNA has higher detection sensitivity and specificity than KK or serum CCA detection [56]. Parasite-specific target sequences can be amplified from cell-free DNA in urine or serum for *S. mansoni*, and from serum, urine or saliva for *S. haematobium* and *S. japonicum* [18]. PCR is also useful is analyzing vaginal lavages which may identify genital schistosomiasis. PCR can also be used to investigate the association between schistosomiasis in women and the increased susceptibility to human immunodeficiency virus (HIV) [57]. Despite its usefulness and high specificity, and the better sensitivity than urine filtration, KK or POC-CCA, its use is still limited in SSA. More recently, loop-mediated isothermal amplification (LAMP) was compared to PCR for the amplification of the 110 bp fragment of a highly repeated 121 bp region of *S. mansoni* and *S. haematobium* 121 bp Dra1 repeats fragment from urine samples. LAMP was found to be as specific as PCR in detecting both schistosome species, as well as being simpler, saves time and does not require the standard thermocycler [58]. It is therefore potentially adaptable to rural conditions as a point-of-care molecular diagnostics kit. Another technique that is currently undergoing research and development is the Recombinase Polymerase Amplification (RPA) assay, which has been demonstrated in *Plasmodium falciparum* and pathogenic *Leptospira* as a reliable genomic DNA based diagnostic [59,60]. The RPA assay has been tested in detecting low levels of *S. haematobium* and *S. japonicum* [61,62]. The RPA assay is rapid, requiring low temperatures, materials that are needed for the assay can be preserved at room temperature and positive reactions are interpreted using lateral flow strips. This assay works through the amplification of the Dra1 DNA region of *S. haematobium*, and it was demonstrated to provide sensitivity as low as 100 fg even when crude urine was spiked in the preparation, suggesting tolerance to inhibitors [61]. RPA has good potential for field application, and further experiments validating their usefulness and bringing down the costs for use in resource limited settings are needed. 

One of the limitations in the field application of DNA based diagnostics is total DNA sample preparation and the purification from urine [61,63]. This has been a bottleneck in the application of genomic DNA based diagnostics, because of the costs, and the technical and time consuming nature of the preparation. RPA, however, offers hope that sample genomic DNA preparation and purification might not be required. Hence, this could potentially bring down the costs of the assay with increased feasibility in a POC setting [61]. Also, discovery of new and better DNA targets for RPA and LAMP analysis needs further investigation.

In most low resource settings, the use of molecular screening methods have been explored; however, due to the limited resources and the requirement of expensive technology, cold chain logistics, uninterrupted power supply, highly skilled manpower, the application of PCR is rare [64,65,66,67]. Improved funding for research and health systems across developing countries would encourage the application of molecular diagnostic tests for screening schistosomiasis.

## 10. Cost Analysis of Diagnostics

In the developing countries, one of the major challenges facing the deployment of most diagnostic methods is the huge costs [68,69]. The drivers of cost of diagnosis include the supply of kits and reagents, labour, the nature of the test (whether multi-step or not), and for field-based diagnosis, transportation is an important factor [70]. Table 1 summarises the diagnostic kits for schistosomiasis and market prices. Microscopy, as a method of schistosomiasis diagnosis, is inexpensive and it does not need extensive training and sophisticated facilities. However, in addition to its low sensitivity for detecting light infections, other limitations include its lack of rapidity, might need a centrifugation or filtration step to concentrate the eggs, as seen in FECT and FLOTAC, thereby increasing the cost for use in resource-poor countries [71]. Kato Katz, although being widely considered to cost about US$2.00 for a single test, to the contrary, a closer cost analysis indicates that the actual cost is much higher than previously documented [72]. Worrell et al. have reported the total cost (including the cost of supplies and labour) of field-based triplicate KK to be approximately $17.54 [70]. This price is very expensive, and it therefore leads to limited application of the test irrespective of its performance. In addition to the cost that is associated with carrying out triplicate KK to enhance sensitivity, the availability of the materials for the protocol can be difficult to obtain in endemic countries except when donated by stakeholders or WHO.

Also, immunoblot assays cost around $3.75 per test or more, which is equally not affordable [75,76]. The current price of CCA test is about $1.75 [45], in comparison with SmCTF-RDT, which costs around $1 per test, which is however not commercially available [74]. Interestingly, it is believed that if CCA dipstick test is internationally accepted as a viable alternative to single stool sample microscopy, the price may drop to a fairly affordable range due to an increase in demand. Moreover, since CCA dipstick is manufactured in Africa, it would be shipped across the continent at a reduced cost [85]. POC-CCA fulfils most of the ASSURED (A = affordable by the affected individuals; S = sensitive; S = specific; U = user-friendly; R = rapid turn-around time and robust performance (e.g., reagents tolerate tropical climate); E = equipment-free; and D = delivered to those in need) criteria for deployment in resource-constrained settings [68]. It is rapid, user-friendly, robust in performance, highly sensitive, and specific [69,86]. Another factor affecting POC-CCA deployment is limited commercial availability, with Rapid Medical Diagnostics, Cape Town, South Africa as the only company producing it at a commercial scale [69]. As it is, without support from governments and stakeholders, the deployment of more sensitive diagnostics at affordable prices, particularly in rural settings of low and middle income countries, remains far fetched [71,86].

## 11. Future Directions

Schistosomiasis diagnosis in most developing countries still relies heavily on microscopy, both for urogenital (urine) and intestinal schistosomiasis (feces). Although this is the approved approach by WHO for urogenital schistosomiasis, a lot of intestinal schistosomiasis cases go undetected in countries that have low transmission rates for intestinal schistosomes, likewise for urogenital schistosomiasis. Affordability of diagnostics that are identified to be very sensitive, such as POC-CCA, is a tough challenge for stakeholders and partners to overcome. Without support from the international community, endemic countries in Africa will be unable to afford kits that cost close to $1 a day for monitoring infections in their communities. This presents a challenge for monitoring the efficacy of control measures being put in place as we march towards the proposed eradication of the parasite by 2020. Efforts towards designing cheaper, more sensitive, and specific RDTs, especially for *S. haematobium*, should be intensified. Lastly, the cost of purchasing the current RDT kit for *S. mansoni* (CCA test kit) needs to be reviewed downwards for wider applicability and use in endemic countries.

## Figures and Tables

**Table 1:** Summary of Available Schistosomiasis Diagnostic Techniques, their Costs, Commercial Availability, and Manufacturers.

Diagnostic Method	Cost of Test Per Sample	Manufacturer	References
**RDTs**			
Hemastix	$0.25	Siemens Healthcare Diagnostics Products Ltd., Llanberis, UK	[73]
SmCTF-RDT	$1	BioGlab Ltd., Nottingham, UK	[72,74]
Immunoblot	$3.75	LD-BIO Diagnostics, Lyon, France	[29,74,75,76]
POC-CCA	$1.75	Rapid Medical Diagnostics, Pretoria, South Africa	[69,77]
UCP-LF CAA	-	-	[78]
ELISA	$1.5	SCIMEDX, New Jersey, USA	[79]
IHA	N/A	Fumouze Laboratories Levallois-Perret, France	[29,79]
**Microscopy**			
Urine filtration	$0.1–0.3	-	[69]
Direct faecal smear	$0.65	-	[69,80]
FECT		-	[69,80]
Kato-Katz	$2.00–2.67	Sterlitech, Kent, Washington, DC, USA	[19,72]
FLOTAC	~$3	University of Naples Federico II, Naples, Italy.	[81]
Mini-FLOTAC	$2.02	University of Naples Federico II, Naples, Italy.	[81,82]
**Molecular diagnostics**			
PCR	$6.4–7.7	Qiagen, Hilden, Germany	[51,82]
LAMP	$0.71–2	New England Biolabs, Hitchin, UK	[83,84]
RPA	$5	TwistDx, Cambridge, UK	[61]

RDT, rapid diagnostic test; UCP-LF CAA, up-converting phosphor-lateral flow circulating anodic antigen (urine-based); ELISA, enzyme-linked immunosorbent assay; IHA, indirect haemagglutination assay; FECT, formalin–ether concentration technique; PCR, polymerase chain reaction; LAMP, loop-mediated isothermal amplification; RPA, recombinase polymerase amplification; POC-CCA, point-of-care circulating cathodic antigen.
